# Pericardial Hydatid Cyst in a Patient With Multisensory Impairments: Case Report and Review

**DOI:** 10.1002/ccr3.71262

**Published:** 2025-10-12

**Authors:** Mohanad Jaber, Nasim Abukaresh, Abedelazeez Mazen Atawneh, Husein Sarahneh, Haya Taha, Haneen Awad, Hossam Salameh

**Affiliations:** ^1^ Clinical Medical Department Faculty of Medicine, Palestine Polytechnic University Hebron Palestine; ^2^ Faculty of Medicine Palestine Polytechnic University Hebron Palestine; ^3^ Emergency Department Hebron Governmental Hospital Hebron Palestine; ^4^ Radiology Department Hebron Governmental Hospital Hebron Palestine; ^5^ Department of Medicine An‐Najah National University Nablus Palestine

**Keywords:** cestode infections, echinococcosis, medistinal cyst, pericardium

## Abstract

A pericardial hydatid cyst (HC) is a rare manifestation of echinococcosis that can present with various clinical features. Management planning depends on the symptoms, involvement, and anatomical features of the HC. Some cases have reported total management with medical management with Albendazole or Mebendazole, while for the vast majority of cases, surgical intervention is needed.

## Graphical abstract

Schematic representation of a pericardial hydatid cyst caused by *Echinococcus granulosus*. The illustration depicts the patient's neurological comorbidities, characteristic multiloculated pericardial cystic lesion on CT imaging, surgical excision under cardiopulmonary bypass, and the zoonotic transmission cycle involving livestock in endemic regions.

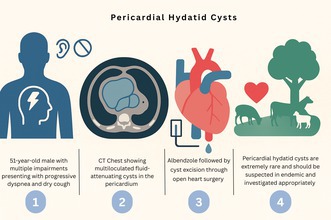

## 1 Introduction

Echinococcosis is an important worldwide parasitic infection endemic in various sheep‐ and cattle‐raising regions [1]. Hydatid cysts (HC) can be found in any part of the body; they most often affect the liver and lungs.

Only 0.5%–2% of cases of hydatid disease involve the heart; involvement of the pericardium is even more uncommon, occurring in only 2%–10% of cases of cardiac hydatid disease [2].

An isolated pericardial HC without myocardial involvement is rare [3].

Pericardial cysts are extremely uncommon in the younger population, with the majority of cases seen post the third decade of life [4].

Presentation is variable and includes non‐specific chest pain, palpitations, and rarely congestive cardiac failure and cardiac tamponade.

Intrapericardial rupture of a HC can lead to life‐threatening complications, such as acute pericarditis with cardiac tamponade and severe anaphylactic shock or compressive effects, which manifest as arrhythmias [2].

Herein, we present the case of a male patient with the diagnosis of an isolated pericardial HC.

## 2 Case History and Exam

A 51‐year‐old male with a known history of epilepsy, managed with valproate and carbamazepine, presented with a 2‐week history of progressive orthopnea and dry cough, which worsened significantly over the past 72 h. The patient is deaf, blind, and intellectually disabled as a sequel to meningitis contracted during early childhood. He is entirely dependent on family support for activities of daily living.

According to family members, he has experienced intermittent episodes of dry cough and nonspecific chest discomfort over the past year. These episodes were previously attributed to mild respiratory infections and did not prompt medical evaluation.

He resides in an area where sheep and livestock rarely pass through, with frequent stray dogs in the vicinity of his home. The family confirmed no presence of a domestic dog. There was no history of recent travel outside the region.

On physical examination, auscultation revealed fine crackles and harsh breath sounds in both lung fields. Cardiac examination revealed normal heart sounds without any additional murmurs or gallops. The remainder of the physical examination was unremarkable.

According to the referral notes, the patient underwent surgical excision of the cyst via median sternotomy under cardiopulmonary bypass. The cyst was difficult to excise intact, and the pericardial cavity was irrigated with hypertonic saline to minimize the risk of recurrence. Albendazole therapy was initiated postoperatively.

A written informed consent was attained from the guardians, and the patient was referred to another hospital to receive appropriate treatment prior to undergoing surgical repair.

## 3 Differential Diagnosis, Investigation, and Treatment

**FIGURE 1:**
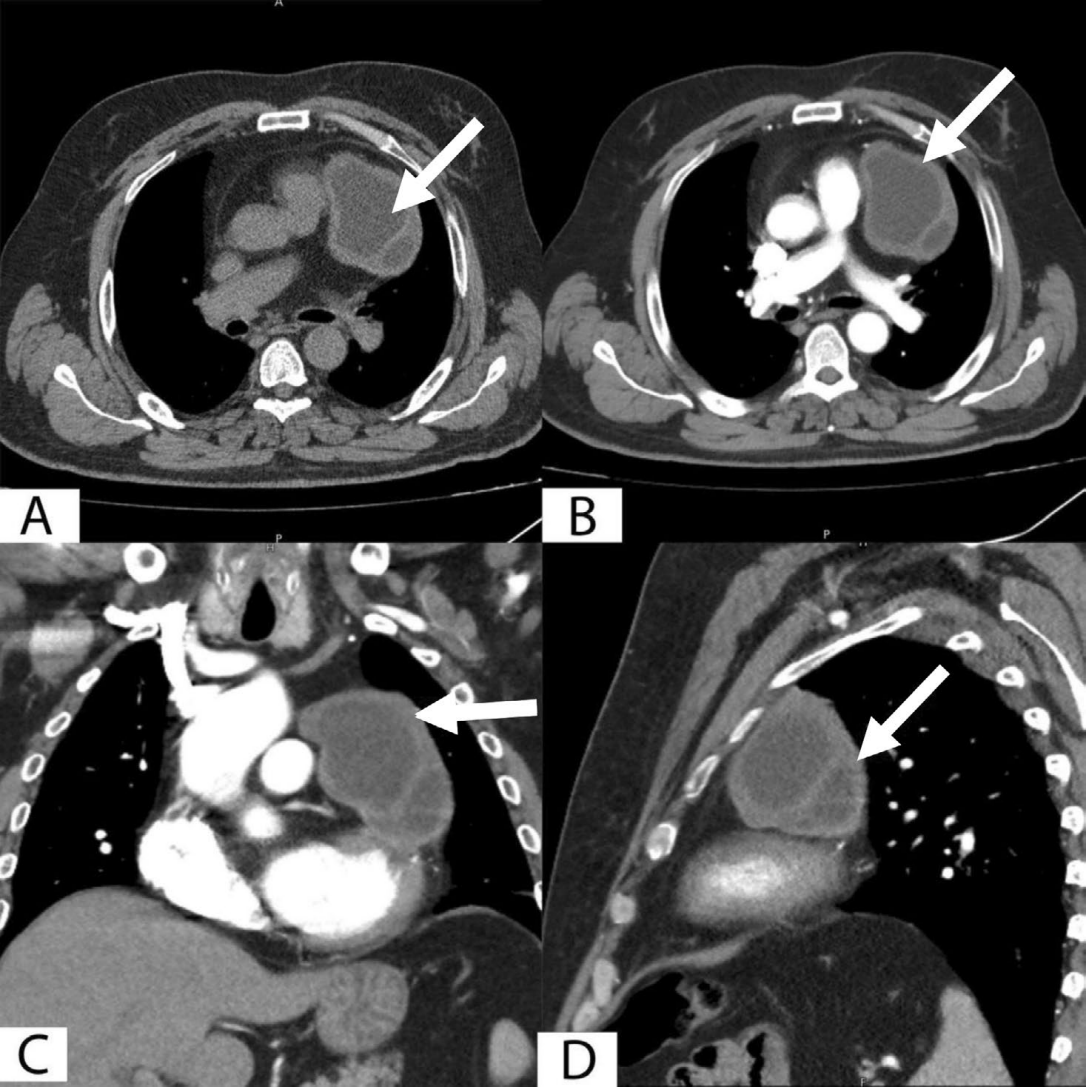
Axial chest CT without iodinated contrast (A) and Multiplanar reconstruction of chest CT imaging with iodinated contrast (B–D) showing a well‐defined mutilocular fluid attenuating nonenhanced lesion (white arrow), located in the left pericardium at the level of the main pulmonary artery, it measures about 8 × 6 cm, most consistent with a hydatid cyst.

Routine laboratory investigations turned out within the normal limits. A chest X‐ray revealed a widened mediastinum, prompting the need for further imaging. A contrast‐enhanced computed tomography (CT) scan of the chest was performed, and the relevant findings are demonstrated in Figure 1.

## 4 Conclusion and Results

After provisional diagnosis, the patient was referred to another hospital to receive appropriate treatment and undergo surgical removal of the cyst. Patients typically are treated with Albendazole for a few months before performing the surgery.

## 5 Discussion

Humans serve as an intermediate host for the parasite Echinococcus granulosus during its larval stage, which can be acquired through the ingestion of contaminated food and water or direct contact with primary hosts, such as dogs [5].

While the parasite most commonly affects the liver and lungs, it can also impact other organs since the larvae can circulate in the blood. This primarily affects rural areas and is regarded as endemic in certain geographical regions like the Middle East, Asia, etc. [6].

Hydatid disease remains endemic in pastoral regions such as the Middle East, North Africa, and parts of Central Asia. Cardiac involvement comprises only 0.5%–2% of all hydatid cases, while isolated pericardial cysts account for just 2%–10% within that small subset [7].

Cardiac HC can very rarely lead to an isolated pericardial HC that can present with cardiac and pulmonary manifestations; however, the disease is most commonly asymptomatic, with symptoms attributed to compression effects. Thus, the size and location of the cyst predominantly determine the clinical picture [8].

A recent retrospective series analyzing 14 cases (2012–2024) reported a mean age of 26 years and noted pericardial involvement in 5 out of 14 patients, reinforcing that pericardial hydatidosis, though rare, may be underrecognized [9].

As illustrated in Flowchart 1, there have been multiple postulations about how the parasite would find its way to the pericardium. The rarer possibility is through hematogenous spread reaching pericardial arterial blood supply, though according to the latest review on 146 cases, more than half of the cases were isolated pericardial HCs, as seen in Table 1, which contradicts the consensus on the rarity of the phenomenon. The second and more likely possibility is perforation in the pericardium either from a primary cardiac HC or HC in other regions like the mediastinum, lung, or other close structures that rupture into the pericardial cavity [9].

**FLOWCHART 1:**
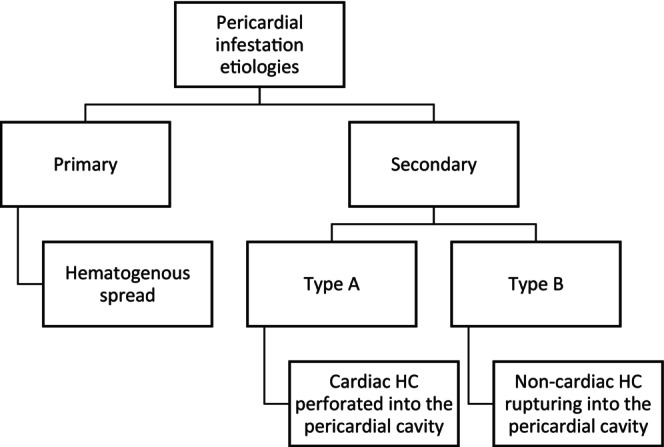
Etiologies of pericardial HCs [9].

**TABLE 1:** Characteristics of patients and clinical manifestations.

Clinical manifestation				
Chest pain 64 (43%)	Dyspnea 53 (36%)	Asymptomatic 14 (10%)	Others (38.2%)	
Distribution of cases among countries				
Turkey 44 (29.72%)	India 27 (18.24%)	Spain 13 (8.78%)	China 10 (6.76%)	Others 54 (≈36.4%)
History of Hydatid disease				
Yes		No		
26 (17.6%)		20 (82.4%)		
Concomitant non‐cardiac HCs				
Yes (44%)		No (56%)		
Involvement in cardiac hydatidosis				
Ventricles (70%)	Pericardium (7%)	Pulmonary artery (6%)	Left atrium (6%)	Interventricular septum (4%)

*Note:* Characteristics of patients and clinical manifestations according to a systematic review of 148 cases of pericardial hydatid cysts [5, 8].

The gradual growth of cardiac HCs allows for a wide range of signs and symptoms, as seen in Table 1, ranging from being asymptomatic in many patients to conditions that have deleterious effects on patient mortality, including arrhythmias, anaphylactic shock, and cardiac tamponade [5], indicating the extensive array of presenting complaints.

Timely diagnosis of pericardial HCs is imperative, as a delay in treatment can lead to detrimental consequences; therefore, imaging techniques were found to be the most accurate diagnostic modalities, like transthoracic echocardiography (TTE), computed tomography (CT), and magnetic resonance imaging (MRI), due to their high sensitivity and ability to uncover much needed detail regarding the spread and involvement of local and even other structures of the body while also providing clearer structural manifestations to differentiate the cyst from other solid structures (fibroma, myxoma) while also evading the possibility of false negative results that can occur with serological testing [1, 10] as demonstrated in Flowchart 2.

**FLOWCHART 2:**
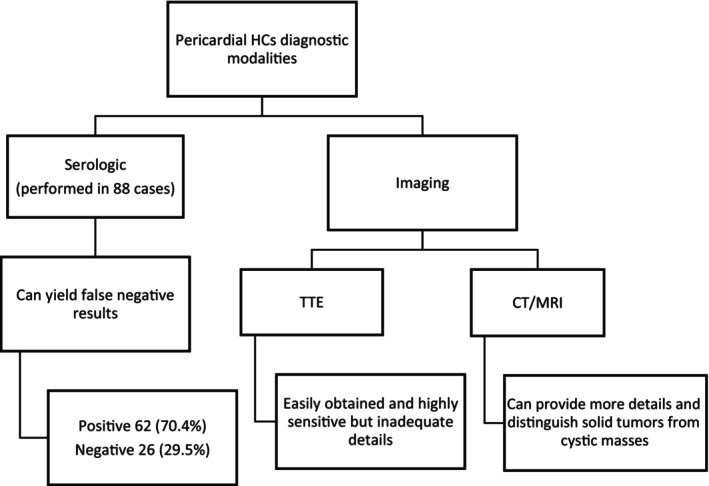
Diagnostic modalities of pericardial HCs [1, 5].

While management of pericardial HCs has been predominantly surgical, three cases have shown that medical treatment can lead to satisfactory results, proven by imaging after long‐term medication with Albendazole [11, 12, 13], especially in patients not fit for surgery or who have extensive involvement of multiple structures, or who are pregnant.

In most cases, open surgery was required for pericardial HCs either through thoracotomy or median sternotomy approaches, with resection being the standard of care, with recommendations of post‐operative medical treatment with Albendazole or Mebendazole to lower the recurrence rate [5].

In some cases, video‐assisted thoracoscopic surgery (VATS) was implemented and has proven to be effective, with satisfactory results. However, it is important to understand that experience and ideal settings are vital when implementing such an intervention instead of open surgery [14, 15].

Given the potential for life‐threatening complications such as tamponade, anaphylaxis, and arrhythmias, early imaging evaluation is crucial in high‐risk patients, even when typical symptoms are obscured by disability. Routine echocardiographic screening may be warranted for echinococcosis patients in endemic regions with high‐risk exposures. A multidisciplinary treatment model combining imaging, surgical excision, and adjunctive anthelmintic therapy offers optimal outcomes.

## Author Contributions

**Mohanad Jaber:** conceptualization, supervision. **Nasim Abukaresh:** conceptualization, data curation, methodology, writing – original draft. **Abedelazeez Mazen Atawneh:** resources. **Husein Sarahneh:** resources. **Haya Taha:** resources. **Haneen Awad:** resources. **Hossam Salameh:** data curation, methodology, visualization, writing – original draft.

## Data Availability

The data that support the findings of this study are available on request from the corresponding author. The data are not publicly available due to privacy or ethical restrictions.
